# Etymologia*: *chikungunya

**DOI:** 10.3201/eid1211.ET1211

**Published:** 2006-11

**Keywords:** etymology, chikungunya

## [chik′′ən-gun′yə]

From the language of the Makonde, northern Mozambique and southeast Tanzania (often misattributed to Swahili), "that which bends up." Chikungunya refers to the stooped posture that develops as a result of arthritic symptoms. A self-limiting disease, chikungunya is caused by an alphavirus spread by the bite of *Aedes* mosquitoes. Though not generally fatal, the disease can be severe. A widespread outbreak in islands of the Indian Ocean, begun in February 2005, has caused >200 deaths.**Sources:** Dorland's illustrated medical dictionary. 30th ed. Philadelphia: Saunders; 2003 and http://www.wikipedia.org

